# Intraconversion of Polar Ginsenosides, Their Transformation into Less-Polar Ginsenosides, and Ginsenoside Acetylation in Ginseng Flowers upon Baking and Steaming

**DOI:** 10.3390/molecules23040759

**Published:** 2018-03-26

**Authors:** Xiang Li, Fan Yao, Hang Fan, Ke Li, Liwei Sun, Yujun Liu

**Affiliations:** 1 College of Biological Sciences and Biotechnology, Beijing Forestry University, Qinghuadonglu No. 35, Haidian District, Beijing 100083, China; 18289743335@163.com (X.L.); Fany_strive@163.com (F.Y.); fwqh1990@163.com (H.F.); like17931@163.com (K.L.); 2 Beijing Beilin Advanced Eco-environmental Protection Technology Institute Co. Ltd., Qinghuadonglu No. 35, Haidian District, Beijing 100083, China

**Keywords:** *Panax ginseng* flower, HPLC, UPLC-QTOF-MS/MS, ginsenosides transformation, acetylation, baking and steaming

## Abstract

Heating is a traditional method used in ginseng root processing, however, there aren’t reports on differences resulting from baking and steaming. Moreover, ginseng flowers, with 5.06 times more total saponins than ginseng root, are not fully taken advantage of for their ginsenosides. Transformation mechanisms of ginsenosides in ginseng flowers upon baking and steaming were thus explored. HPLC using authentic standards of 20 ginsenosides and UPLC-QTOF-MS/MS were used to quantify and identify ginsenosides, respectively, in ginseng flowers baked or steamed at different temperatures and durations. Results show that baking and steaming caused a 3.2-fold increase in ginsenoside species existed in unheated ginseng flowers (20/64 ginsenosides) and transformation of a certain amount of polar ginsenosides into numerous less polar ginsenosides. Among the 20 ginsenosides with standards, polar ginsenosides were abundant in ginseng flowers baked or steamed at lower temperatures, whereas less polar ginsenosides occurred and were enriched at higher temperatures. Furthermore, the two types of heating treatments could generate mostly similar ginsenosides, but steaming was much efficient than baking in transforming polar- into less polar ginsenosides, with steaming at 120 °C being comparably equivalent to baking at 150 °C. Moreover, both the two heating methods triggered ginsenoside acetylation and thus caused formation of 16 acetylginsenosides. Finally, a new transformation mechanism concerning acetyl-ginsenosides formation was proposed.

## 1. Introduction

*Panax ginseng* Meyer, has been utilized in China, Korea and Japan for thousands of years as a medicinal plant, and it is one of the most popular herbal medicines used as a dietary supplement in recent years [1]. The main bioactive compositions of *P. ginseng* and several other *Panax* species are triterpene saponins, termed ginsenosides, which are considered to be responsible for a variety of pharmacological effects, such as anti-inflammatory, antioxidant, anti-tumor, and anti-aging activities [2,3]. Up to now, more than 150 ginsenosides have been detected in the genus *Panax* [4]. According to their aglycone structures, ginsenosides are divided into three groups: dammaranes, ocotillols, and oleananes. The dammarane triterpenes to which most ginsenosides belong can be generally further classified as protopanaxadiols (e.g., Rb_1_, Rb_2_, Rb_3_, Rc, Rd, Rg_3_, and Rh_2_) and protopanaxatriols (e.g., Re, Rf, Rg_1_, Rg_2_ and Rh_1_) [5,6]. Individual saponins possess various pharmacological activities. For instances, the ginsenoside Rg_3_ showed the ability to suppress tumor growth and tumor angiogenesis [7], and the ginsenoside CK (i.e., compound K) exhibits direct cytotoxic and growth-inhibitory effects against tumor cells [8].

In Asia, there are two types of commercial ginseng root available in herbal medicine markets: white ginseng and red ginseng. White ginseng is produced by dehydration of the fresh ginseng root under direct sunlight, and red ginseng is manufactured by steaming the fresh ginseng root at 95–100 °C for 2–3 h one to five or six times, and then drying under sunlight [9]. It is well known that red ginseng shows better bioactive potential than white ginseng does [10]. Many articles have reported that changes in the chemical structures of ginsenosides induced by steaming significantly improve the biological activities of the ginseng root. During the steaming process, the amount of polar ginsenosides decreases, and that of less polar ginsenosides increases [3]. Due to its relatively high levels of the less polar ginsenosides, red ginseng exhibits much more potent anticancer, antidiabetic and anti-inflammatory bioactivities [11,12]. Generally, steaming exerts two physiochemical effects, i.e., hydration and high-temperature exposure (and high-pressure at temperatures higher than 100 °C), but whether both (or the three) effects could lead to changes in the composition of ginsenosides is not clear. Compared with steaming, baking exerts exposes ginsenosides to high-temperature. Hence, comparative analysis of the effects on ginseng root as well as other parts of *P. ginseng* of baking and steaming treatments would help us to explore which of the two (or three) physiochemical effects can lead to changes of ginsenosides, and to further deduce related mechanisms of intraconversion of polar ginsenosides and their transformation into less polar ginsenosides.

In Traditional Chinese Medicine, ginseng root is the most commonly used part of the herb [13]. Other parts of *P. ginseng*, especially the flowers, also have many kinds of pharmacological activities, including immunity-enhancing [13], anti-tumor [14], antifatigue, and anti-aging effects [15]. For a long time, ginseng flowers have been exploited extensively in the form of drugs and/or foods. For instance, they are utilized in several famous Traditional Chinese Medicine formulae, and have long been used in China as an exhilarant or tonic in the form of a health tea [16]. Modern pharmaceutical studies also indicated that the pharmacological value of ginseng flower is much better than that of ginseng root. In fact, the total saponin content of ginseng flowers is 5.06 times that of ginseng root, thus they could be employed as an alternative source of ginsenosides and supplementary ingredients [17]. Previously, a few publications have focused on steamed ginseng flowers. Chol et al. [18] reported that dammarane-type ginsenosides inhibit growth of HepG2 and SK-Hep1 cells. Tung et al. [19] documented that ginsenosides from steamed flowers show inhibitory effect in bone marrow-derived dendritic cells. Nevertheless, none of the reports carried out any comparison of the differences in the transformations of ginsenosides between baked and steamed ginseng flowers.

The ongoing development in ultrahigh-performance liquid chromatography (UPLC) coupled with MS-based metabolomics exhibits advantages of high resolution, selectivity and sensitivity, and allows rapid analyses of components from complex medicinal herb mixtures [20]. Zhou et al. [21] developed a method for simultaneous quantification of 21 ginsenosides from red ginseng. Wang et al. [22] detected a total of 131 ginsenosides from cultivated and forest ginsengs and further quantified 19 representative ginsenosides. Xu et al. [20] identified chemical markers which could be used to illustrate the transformation of ginsenosides in processed red ginseng. Anyway, most of these articles investigated the steamed ginseng root and analyzed its ginsenoside changes, and few of them focused on other parts of the ginseng plant, including ginseng flowers, and none of them explored the mechanism of transformation of ginsenosides upon baking, although baking, in addition to steaming, is in practice also a popular measure for processing ginseng. In the present study, we investigated the effects of baking and steaming at different temperatures and for various durations on the saponin composition of ginseng flowers utilizing the UPLC-QTOF-MS/MS technique and further quantified 20 representative ginsenosides by HPLC. These explorations should allow us to have a better understanding of the subtle differences between the two types of heated ginseng flowers, and to examine the major structural changes of ginsenosides during baking and steaming.

## 2. Results and Discussion

### 2.1. Effects of Baking and Steaming on HPLC Profiles of Ginsenosides in Ginseng Flowers

**Figure 1:**
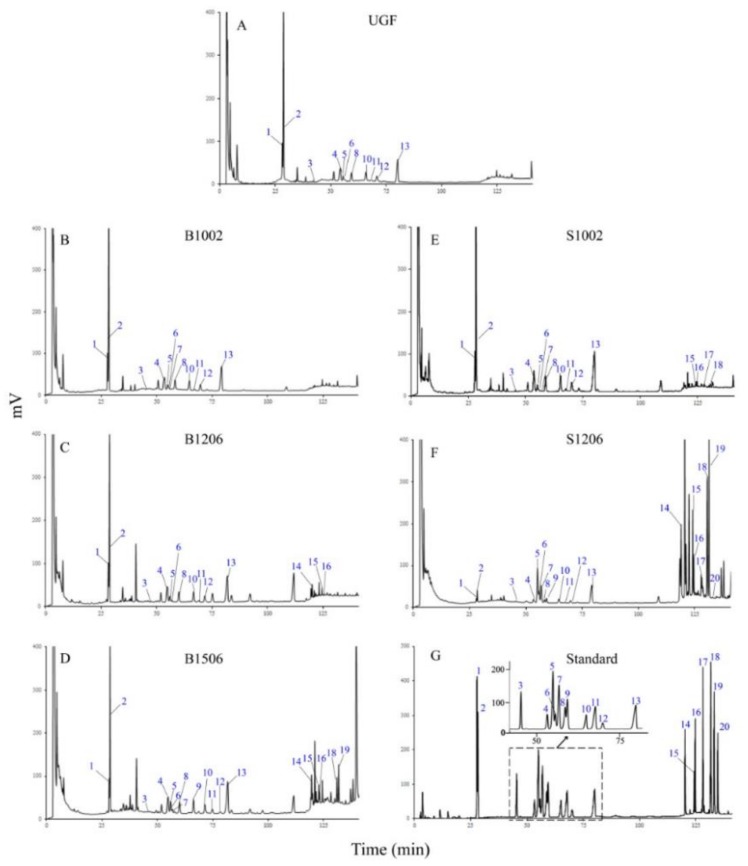
HPLC analyses of ginsenosides in unheated and heated ginseng flowers. Shown here are chromatograms of unheated ginseng flowers (UGF; (**A**)); flowers baked for 2 h at 100 °C (B1002; (**B**)); 120 °C (B1206; (**C**)) and 150 °C (B1506; (**D**)); and flowers steamed for 2 h at 100 °C (S1002; (**E**)) and for 6 h at 120 °C (S1206; (**F**)); and standards of the 20 ginsenosides (**G**). Ginsenosides marked are: (1) Rg_1_, (2) Re, (3) Rf, (4) Rb_1_, (5) 20(*S*)-Rg_2_, (6) 20(*S*)-Rh_1_, (7) 20(*R*)-Rg_2_, (8) Rc, (9) 20(*R*)-Rh_1_, (10) Rb_2_, (11) Rb_3_, (12) F_1_, (13) Rd, (14) F_4_, (15) 20(*S*)-Rg_3_, (16) 20(*R*)-Rg_3_, and (17) 20(*S*)-PPT, (18) Rg_5_, (19) CK, and (20) Rh_2_. Peak numbers of the 20 standard ginsenosides not shown were not detected in the corresponding flower samples. An inset within Figure 1G is an amplification of the partial profile between 40 and 85 min as indicated by the dashed box.

A HPLC-UV method was used for analyzing the ginsenosides of unheated ginseng flowers (UGF) and heated ginseng flowers. Compared with that of UGF (Figure 1A), the HPLC profiles of all heated flowers changed to different extents (Figure 1B–F).

The profile of flowers baked at 100 °C for 2 h (B1002; Figure 1B) was very similar to that of UGF in which ginsenosides appeared at retention times between 25 and 90 min (polar ginsenosides) as well as between 110 and 140 min (less polar ginsenosides), indicating that the treatment of B1002 did not involve much transformation of ginsenosides. When ginseng flowers were baked at 120 °C for 6 h (B1206; Figure 1C), the profile changed to a certain extent: numbers and individual peak areas of the polar ginsenosides between 25 and 90 min were increased and changed, respectively, with more kinds and amounts of less polar ginsenosides occurring but still at relatively lower concentrations between 110 and 140 min. When ginseng flowers were baked at 150 °C for 6 h (B1506; Figure 1D), however, the profile changed to a great extent: numbers and most individual peak areas of the polar ginsenosides between 25 and 90 (100) min were further increased, and those of the less polar ginsenosides between 110 and 140 min were increased. The results demonstrated that baking at a high temperature and/or for a longer period could lead to polar ginsenosides to be transformed into less polar ginsenosides.

Compared with the baking results described above, steaming, except for providing a high temperature, exerts an extra influence by creating a high moisture environment as well as a high pressure when the temperature is higher than 100 °C. Thus, when ginseng flowers were steamed at 100 °C for 2 h (S1002; Figure 1E), the profile changed to a greater extent compared to those of UGF (Figure 1A) as well as to those baked at 100 °C for 2 h (Figure 1B). Namely, numbers and individual peak areas of both the polar ginsenosides between 25 and 90 min and the less-polar ginsenosides between 110 and 140 min were increased and changed, respectively (Figure 1E), and the increases and changes were even more apparent than those that occurred in flowers baked at 120 °C for 6 h (Figure 1C). A more striking contrast is exhibited in Figure 1F, in which flowers steamed at 120 °C for 6 h (S1206) show a profile of obviously decreased polar ginsenosides between 25 and 90 min and dramatically increased less polar ginsenosides between 110 and 140 min, implying that significant transformation from polar into less polar ginsenosides must be occurring during this steaming treatment.

Finally, by comparing the HPLC profiles of flowers in the treatments of S1002 (Figure 1E) and S1206 (Figure 1F) with those of B1206 (Figure 1C) and B1506 (Figure 1D), respectively, it is easy to figure out that they are similar in pairs, suggesting that the effect of high baking temperature could be compensated by providing a high moisture and pressure environment at a relatively lower steaming temperature.

### 2.2. Effects of Baking and Steaming on 20 Representative Ginsenosides

#### 2.2.1. Changes in Total Amount of the 20 Ginsenosides upon Baking and Steaming

**Figure 2:**
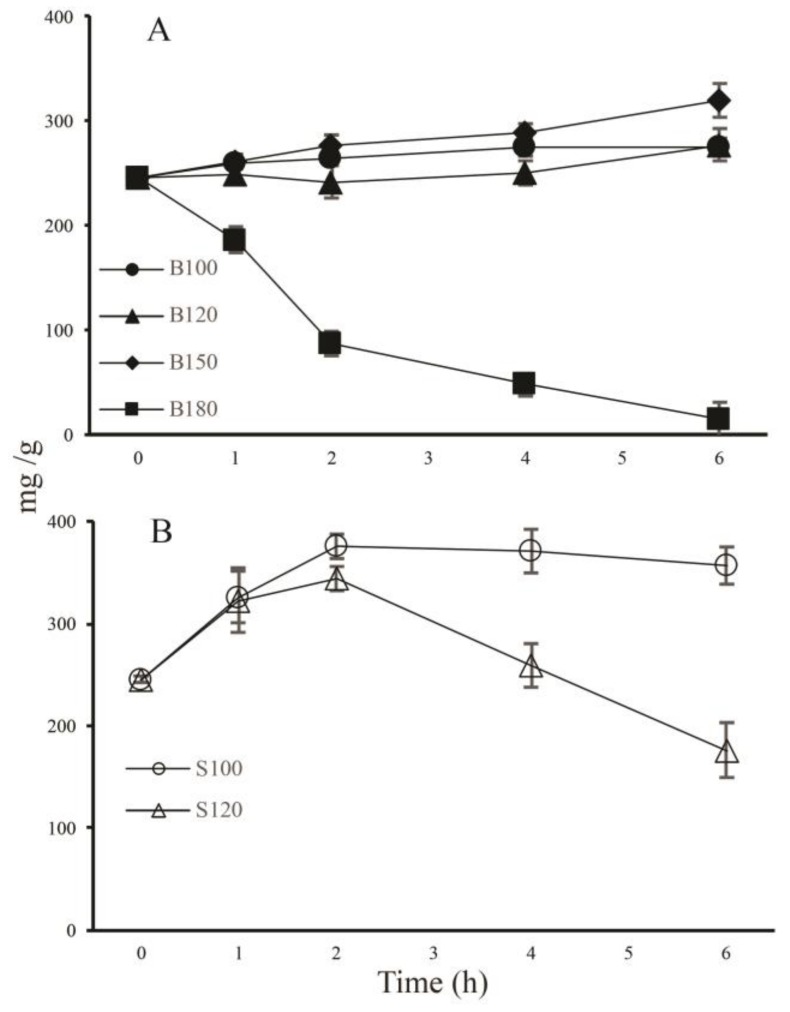
Total contents of 20 ginsenosides in unheated and heated ginseng flowers. (**A**) Ginseng flowers baked at 100 (B100), 120 (B120), 150 (B150) and 180 (B180) °C for (0)1–6 h; (**B**) Ginseng flowers steamed at 100 (S100) and 120 (S120) °C for (0)1–6 h. Data at 0 h represent those of unheated ginseng flowers (UGF). Values are denoted as the mean ± standard deviation (*n* = 3).

In order to quantitatively analyze the ginsenosides, we established a HPLC method that could simultaneously determine 20 representative ginsenosides. Figure 1G shows a HPLC profile of authentic standards of the 20 ginsenosides which were well separated and were also unambiguously identified in each of the flower samples (Figure 1A–F). The total amount of the 20 ginsenosides was accordingly calculated for each of the heated flowers (Figure 2). As shown in Figure 2A, total contents of the 20 ginsenosides upon baking were increased slightly and almost parallely at 100, 120 and 150 °C within the 6 h treatments, suggesting that while transformation of polar ginsenosides into more less polar ginsenosides occurred under these baking treatments (see Figure 1A–D), the total amount of the 20 ginsenosides did not decline (In fact, it was slightly increased). A dramatic and near linear decrease in the total content of the 20 ginsenosides occurred in flowers baked at 180 °C, and the content decreased almost to zero at 6 h, indicating that most of the ginsenosides were damaged by this high temperature baking process.

On the other hand, as shown in Figure 2B, the total contents of the 20 ginsenosides increased significantly to the exactly the same extent upon steaming at 100 and 120 °C during the first hour, and they further increased differently up to 2 h, with the total content in flowers steamed at 100 °C being much higher than that at 120 °C.

The total contents then declined differently until 6 h, with the decrease rate at 120 °C being much higher than that at 100 °C. The results also demonstrated that in view of the increase in total content of the 20 representative ginsenosides, which will be discussed further below, the effect of steaming was much better than that of baking at the same temperature, and the optimal steaming temperature and duration were 100 °C and 2 h, respectively.

Based on the above results, it is obvious that total amount of the 20 ginsenosides with authentic standards increased slightly upon baking (Figure 2A) and significantly upon steaming (Figure 2B), the possible reason was that contents of the 20 ginsenosides with baking treatments changed slowly and those with steaming treatments rapidly and efficiently due to different influences from the water vapor. Moreover, when flowers were baked at 180 °C, numerous ginsenosides were damaged and the content was almost reduced to zero, during which the Maillard reaction might be involved, numerous aromas are generated, and most of the ginsenosides decomposed at high temperatures [23,24,25]. Furthermore, where did these increased amounts of ginsenosides come from? We assume that the increase in total amount of the 20 ginsenosides was the result of transformations of other ginsenosides for which we had no authentic standards (possibly malonylginsenosides) [20].

#### 2.2.2. Transformation of Polar Ginsenosides into Less Polar Ginsenosides upon Baking and Steaming

**Figure 3:**
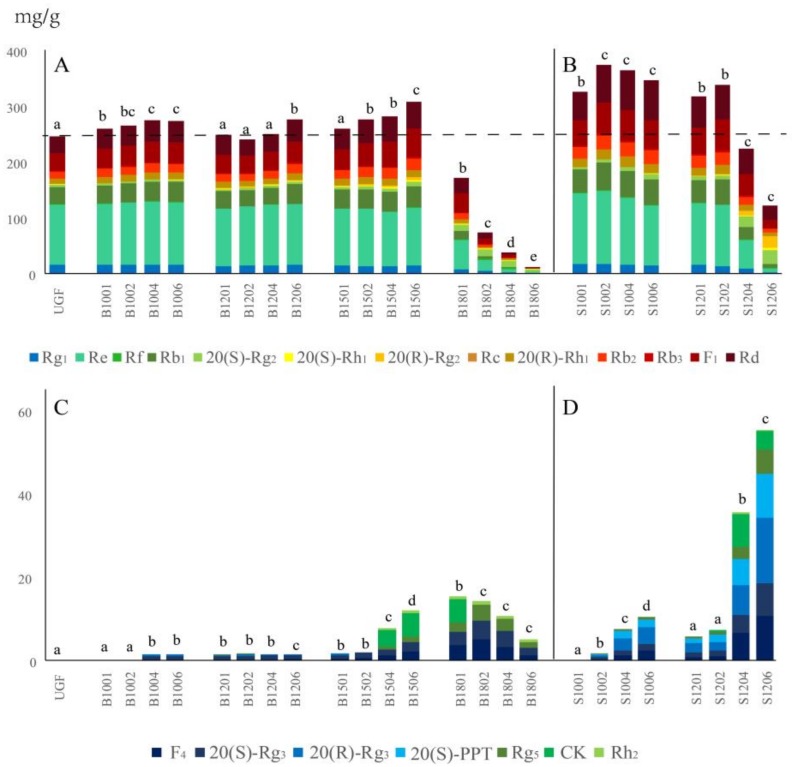
Changes in contents of polar and less-polar ginsenosides in heated ginseng flowers. Shown are contents of polar (**A**,**B**) or less-polar (**C**,**D**) ginsenosides, as indicated by differently-colored columns, in ginseng flowers with various regimes of baking ((**A**) or (**C**)) and steaming ((**B**) or (**D**)). Values marked by the same letter are not significantly different (*p* > 0.05), and those marked by different letters at same temperatures are significantly different (*p* < 0.05) to those of unheated ginseng flowers (UGF).

To further characterize the transformation of ginsenosides during the two types of heating, the 20 ginsenosides were divided into two groups, namely, 13 polar ginsenosides including Rg_1_, Re, Rf, Rb_1_, 20(*S*)-Rg_2_, 20(*S*)-Rh_1_, 20(*R*)-Rg_2_, Rc, 20(*R*)-Rh_1_, Rb_2_, Rb_3_, F_1_ and Rd (Figure 3A,B), which are usually relatively less bioactive and appeared between 25 and 90 min in the HPLC profiles (Figure 1), and seven less polar ginsenosides including F4, 20(*S*)-Rg3, 20(*R*)-Rg3, 20(*S*)-PPT, Rg5, CK and 20(*S*)-Rh2 (Figure 3C,D), which are usually more bioactive and appear between 110 and 140 min (Figure 1).

It is worth noting that contents of individual ginsenosides after the various baking or steaming treatments, in addition to their total contents, were also different as indicated by the color gradient in the individual columns of Figure 3. In UGF, only the 13 polar ginsenosides were detected (Figure 3A,C) and they ranked in contents as Re > F_1_ > Rb_1_ > Rd > Rg_1_ > Rb_2_ > Rc > 20(*S*)-Rg_2_ > Rb_3_ > Rf > 20(*S*)-Rh_1_ > 20(*R*)-Rg_2_ (Figure 3A). Total contents of the 13 polar and seven less polar ginsenosides were the highest in B1506 (Figure 3A) and B1801 (Figure 3C) among all the baking treatments and they ranked in contents as Re > F_1_ > Rd > Rb_1_ > Rb_2_ > Rc > Rg_1_ > 20(*S*)-Rg_2_ > 20(*R*)-Rg_2_ > Rb_3_ > 20(*S*)-Rh_1_ > Rf > 20(*R*)-Rh_1_, and CK > 20(*S*)-Rg_3_ > F_4_ > Rg_5_ > Rh_2_ > 20(*R*)-Rg_3_ = 20(*S*)-PPT, respectively. In contrast, among all the steaming products, the total contents of the 13 polar and seven less polar ginsenosides were the highest in S1002 (Figure 3B) and S1206 (Figure 3D), and they ranked in contents as Re > Rd > F_1_ > Rb_1_ > Rb_2_ > Rg_1_ > Rc > 20(*S*)-Rg_2_ > Rb_3_ > Rf > 20(*R*)-Rg_2_ = 20(*S*)-Rh_1_ = 20(*R*)-Rh_1_, and 20(*S*)-Rg_3_ > 20(*R*)-Rg_3_ > 20(*S*)-PPT > Rg_5_ > CK = F_4_ = Rh_2_, respectively.

Comparing the total content of the 13 polar ginsenosides of UGF, it increased slightly up to 4 h then remained steady during the next 2 h upon baking at 100 °C, exhibiting little changes with no statistical difference up to 4 h, and then increased greatly at 6 h upon baking at 120 °C, and increased much more rapidly until 6 h upon baking at 150 °C. However, the total content of these 13 polar ginsenosides decreased dramatically and was almost zero at 6 h upon baking at 180 °C (Figure 3A). On the other hand, the total content of the 13 polar ginsenosides upon steaming at 100 °C increased and reached the highest value at 2 h (about 1.52 times that of UGF), then it decreased steadily until 6 h, but those of the four steaming treatments were much higher than that of UGF. Total content of these 13 polar ginsenosides upon steaming at 120 °C increased at the first 2 h then decreased more abruptly than that at 100 °C, making the total contents at 4 and 6 h much lower than that of UGF as indicated by the dashed horizontal line (Figure 3B).

Figure 3C,D show the changes in total contents of less polar ginsenosides caused by the two types of heating. The seven less polar ginsenosides, which were not detected in UGF as well as in ginseng flowers upon baking at 100 °C for 1 h (B1001) and 2 h (B1002), were newly generated, but with no significant differences, upon baking at 100 °C for 4 and 6 h (B1004 and B1006), at 120 °C for 1, 2, 4 and 6 h (B1201–B1206), and at 150 °C for 1 and 2 h (B1501 and B1502). The content increased rapidly upon baking at 150 °C for 4 and 6 h (B1504 and B1506) and at 180 °C for 1 h (B1801), then decreased with time at this highest baking temperature (B1802–B1806) (Figure 3C). Regarding steaming treatments, the seven less polar ginsenosides were not detected at 100 °C for 1 h (S1001), newly occurred at 100 °C for 2 h (S1002), and increased up to 6 h (S1004 and S1006). When ginseng flowers were steamed at 120 °C, the seven ginsenosides appeared at 1 h (S1201) and increased abruptly from 2 to 6 h (S1202–S1206) (Figure 3D). The results indicate that both baking and steaming could result in transformation of polar to less polar ginsenosides. Moreover, while baking at the highest temperature of 180 °C led to certain degree generation of less polar ginsenosides (Figure 3C), it also resulted in degeneration of a large amount of polar ginsenosides (Figure 3A). On the contrary, polar ginsenosides in flowers steamed at the higher temperature of 120 °C decreased in a less extent (Figure 3B) but the active less polar ginsenosides increased significantly (Figure 3D).

Above all, during the heating processes of ginseng flowers, polar ginsenosides could be converted to less polar ginsenosides, and high temperature (and/or pressure) and prolonged heating period accelerated these transformations, especially in steaming treatments (see Figure 3). For example, Re, the highest content ginsenoside in UGF (Figure 3A) decreased drastically upon baking at 180 °C (Figure 3A) and steaming at 120 °C (Figure 3B). Similar trends of changes in contents after heating occurred in other polar ginsenosides, including Rg_1_, Rf, Rb_1_, Rc, Rb_2_, Rb_3_, F_1_ and Rd (Figure 3A,B). The baking treatment at 180 °C might involve the so-called Maillard reaction and lead to degradation of the ginsenosides, and steaming treatment at 120 °C, as previously reported [26], might cause transformation into numerous less polar ginsenosides. On the other hand, F_4_, which was not detected in UGF, became the highest less polar ginsenoside in content upon steaming (Figure 3D). Similar trends of changes in contents after heating also occurred in other less polar ginsenosides, including 20(*S*)-Rg_3_, 20(R)-Rg_3_, CK, 20(*S*)-PPT and Rg_5_ (Figure 3C,D).

### 2.3. Identification of Ginsenosides in Ginseng Flowers Using UPLC-QTOF-MS/MS

To characterize and compare saponin compositions in ginseng flowers without and with heating described above, extracts of UGF, B1206, B1506, S1204 and S1206 were analyzed using UPLC-QTOF-MS/MS in a negative ion mode.

As the UPLC chromatogram at 203 nm of Supplementary Figure S2 and data of Table 1 show, a total of 64 ginsenosides were detected. To be specific, 20 were detected in UGF (Supplementary Figure S2B), 33 and 47 from B1206 and B1506 (Supplementary Figure S2C,D), and 40 and 41 from S1204 and S1206 (Supplementary Figure S2E,F), respectively. By analyzing the MS data of the 64 ginsenosides, including retention time (TR), experimental *m*/*z*, molecular formula, error of the experimental *m*/*z*, and MS^2^ fragments of individual compounds, these compounds were successfully identified through referring to available literatures as well as the MassBank MS database (http://www.massbank.jp/en/database.html). Furthermore, 20 of them, i.e., Rg_1_, Re, Rf, Rb_1_, 20(*S*)-Rg_2_, 20(*S*)-Rh_1_, 20(*R*)-Rg_2_, Rc, 20(*R*)-Rh_1_, Rb_2_, Rb_3_, F_1_, Rd, F_4_, 20(*S*)-Rg_3_, 20(*R*)-Rg_3_, 20(*S*)-PPT, Rg_5_, CK and Rh_2_ were additionally confirmed by comparing their mass fragment ions and retention times with those of their corresponding authentic standards (Supplementary Figure S2A). As shown in Table 1, the 64 identified ginsenosides could be classified into two groups, i.e., those that originally existed in UGF (20 ginsenosides; data colored in black) and those that occurred after heating treatments (44 ginsenosides; data colored in blue). Obviously, more than two thirds of the 64 ginsenosides were newly generated upon heating.

#### 2.3.1. Ginsenosides Originally Existing in UGF

The 20 ginsenosides originally existing in UGF (Supplementary Figure S3B) included one that disappeared upon heating that was identified as acetyl-Re isomer V (Compound **29** of Table 1). The other 19 ginsenosides were identified as Rg_1_, Re, Floral G-P, Floral G-P isomer, Re_1_ (or one of Re_2_, Re_3_, and NG-N isomer), acetyl-Re, Rf, NG-R_2_, F_3_, F_5_, Rb_1_, 20(*S*)-Rg_2_, Rc, Rb_2_, Rb_3_, F_1_, Rd, acetyl-Rd and NG-Fe, respectively (Table 1, data in black). Moreover, most of them were polar ginsenosides and existed at high levels in UGF as well as heated ginseng flowers, indicating that they possessed relatively good thermal stability. According to previous reports [13,26,27,28], malonylginsenosides are a type of natural ginsenosides that exists in fresh ginseng flower which can be detected by mass spectrometry. However, malonylginsenosides were not found in our results, and instead we detected three acetylginsenosides (acetyl-Re, acetyl-Re isomer V and acetyl-Rd) in UGF. Therefore, we speculate that the malonylginsenosides in fresh ginseng flowers underwent degradation during the natural drying process by losing carbon dioxide and producing acetyl ginsenosides. Furthermore, like malonylginsenosides, these three acetylginsenosides with no standards might be deacetylated and transformed to their corresponding ginsenosides with standards in the present study, which might also partially explain the increase of total amount of the 20 ginsenosides mentioned above (Figure 2 and Figure 3).

#### 2.3.2. Ginsenosides Newly Generated in Heated Ginseng Flowers

The 44 ginsenosides newly generated in heated ginseng flowers (Supplementary Figure S2C–F; Table 1, data colored in blue) could be further classified into the following three subgroups:

Ten ginsenosides occurred solely after steaming at 120 °C for 4 h (S1204) and/or 6 h (S1206), and they were identified as G-Ia, Re isomer, 20(*R*)-Rg_2_, 20(*R*)-Rh_1_, Rk_3_, Rg_3_ isomer, G-La, 20(*S*)-PPT, 20(*R*)-PPT and acetyl-Rg_3_ isomer, respectively (Table 1, ginsenosides numbered in yellow color at the leftmost column). Among these ten ginsenosides, four, i.e., 20(*R*)-Rg_2_, 20(*S*)-PPT, 20(*R*)-PPT and acetyl-Rg_3_ isomer (Table 1; Compounds **22**, **54**, **55** and **58**), were detected both in S1204 and S1206, indicating that they were transformed easily with good thermal stability at 120 °C, and the other five (Table 1; Compounds **3**, **4**, **24**, **49** and **51**) were detected only in S1206, implying that transformation of these ginsenosides was difficult and thus required a relatively higher temperature. Furthermore, Rg_3_ isomer (Table 1; Compound **50**) was only detected in S1204, indicating that it was transformed easily and had relatively poor heat stability.

Seventeen ginsenosides occurred solely after baking for 6 h at 120 °C (B1206) and/or 150 °C (B1506), and they were identified as acetyl-Re isomer I, acetyl-Re isomer II, acetyl-Re isomer III, acetyl-Rg_1_ I isomer, acetyl-Re isomer IV, acetyl-Rg_1_ II, PQ-R_1_, acetyl-Rb_2_ isomer, Rs_1_ (or Rs_2_), acetyl-Rg_3_ isomer, PQ-R_1_ isomer, acetyl-Rb_2_, acetyl-Rd isomer I, acetyl-Rb_3_, acetyl-Rd isomer II, acetyl-Rd isomer III and Rg_6_ isomer, respectively (Table 1, ginsenosides numbered in red at the leftmost column). All of them could be transformed through only baking and had nothing to do with whether water and/or pressure was involved or not. Among them, only PQ-R_1_ isomer (Table 1, Compound **36**) occurred solely in B1206, indicating that this ginsenoside was easily transformed with poor heat stability, while another seven ginsenosides, i.e., acetyl-Re isomer II, Rs_1_ (or Rs_2_), acetyl-Rb_2_, acetyl-Rb_3_, acetyl-Rd isomer I, acetyl-Rd isomer II and acetyl-Rd isomer III (Table 1, Compounds **8**, **33** or **35**, **38**, **39**–**41** and **44**), were detected in both B1206 and B1506, implying that they were easily transformed and possessed good thermal stability. The rest nine ginsenosides (Table 1, Compounds **6**, **9**–**11**, **14**, **25**, **31**, **34** and **45**) were only detected in B1506, indicating that these ginsenosides were difficult to convert and exhibited the highest thermostability upon baking at higher temperature.

The other seventeen ginsenosides occurred after both steaming and baking at 120 °C and/or 150 °C for 4 h (S1204) and/or 6 h (S1206, B1206, and B1506), and they were identified as F_3_ (or F_5_), 20(*S*)-Rh_1_, PQ-R_1_ isomer, Rs_1_ (or Rs_2_), Rg_6_, Pseudo-Rc1, F_4_, 20(*S*)-Rg_3_, 20(*R*)-Rg_3_, acetyl-20(*S*)-Rg_3_, acetyl-20(*R*)-Rg_3_, Rk_1_, Rg_5_, CK, Rh_2_, Rs_5_ and Rs_4_, respectively (Table 1; ginsenosides numbered in brown at the most left column). Among them, only F_3_ (or F_5_; Table 1, Compound **19**) appeared in S1204 and both the baking treatments (B1206 and B1506), indicating that it was more stable to baking treatment, and a longer steaming period of 6 h led it to be degenerated. Two ginsenosides, 20(*S*)-Rh_1_ and 20(*R*)-Rg_3_ (Table 1, Compounds **21** and **53**), were detected in B1206 and both steaming treatments (S1204 and S1206), suggesting that these two ginsenosides were more stable to steaming treatment, and the baking treatment at higher temperature of 150 °C led them to be degraded. Eight ginsenosides, i.e., Rg_6_, acetyl-20(*S*)-Rg_3_, acetyl-20(*R*)-Rg_3_, Rg_5_, CK, Rh_2_, Rs_5_ and Rs_4_ (note: most of them are less polar ginsenosides; Table 1, Compounds **46**, **56**, **57** and **60**–**64**), were detected in B1506 and the two steaming treatments (S1204 and S1206), suggesting that these ginsenosides were stable to steaming treatments and in the baking treatment at the higher temperature (150 °C). The results further indicated that these less polar ginsenosides need more energy and/or higher moisture when they are transformed and these eight ginsenosides might be the final products of transformation. The remaining six ginsenosides (Table 1, Compounds **30**, **35**, **47**, **48**, **52** and **59**) were detected in all four heating treatments (i.e., S1204, S1206, B1206 and B1506), indicating that these ginsenosides were generated upon simple heating.

In addition, to further examine the differences between steaming and baking, we compared the data of S1206 with those of B1206. Except for the 23 compounds detected in both treatments (Table 1, Compounds **1**, **2**, **12**, **13**, **15**–**18**, **22**, **23**, **26**–**28**, **30**, **32**, **35**, **42**, **46**–**48**, **52**, **53** and **59**), seven ginsenosides [floral G-P, acetyl-Re isomer II, F_5_ (or F_3_), Rs_1_ (or Rs_2_), acetyl-Rb_2_, acetyl-Rd isomer I and acetyl-Rb_3_] were detected only in B1206 (Table 1, Compounds **5**, **8**, **19**, **33** and **38**–**40**), and eighteen [G-Ia, Re isomer, 20(*R*)-Rg_2_, 20(*R*)-Rh_1_, NG-Fe, Rg_6_, Rk_3_, G-La, 20(*S*)-PPT, 20(*R*)-PPT, acetyl-20(*S*)-Rg_3_, acetyl-20(*R*)-Rg_3_, acetyl-Rg_3_ isomer, Rg_5_, CK, Rh_2_, Rs_5_ and Rs_4_] only in S1206 (Table 1, Compounds **3**, **4**, 22, **24**, **43**, **46**, **49**, **51**, **54**–**58** and **60**–**64**). Obviously, under the same temperature and duration, more ginsenosides were detected upon steaming and most of them were less polar ginsenosides, and many fewer ginsenosides were detected upon baking. However, similar types of ginsenosides were found in both S1206 and B1506 (Table 1, Compounds **1**, **2**, **12**, **13**, **15**–**18**, **20**, **23**, **26**–**28**, **30**, **32**, **35**, **42**, **46**–**48**, **52**, **56**, **57** and **59**–**64**), implying further that the higher temperature of baking was required for transforming similar types of ginsenosides than in steaming treatment.

**Figure 4:**
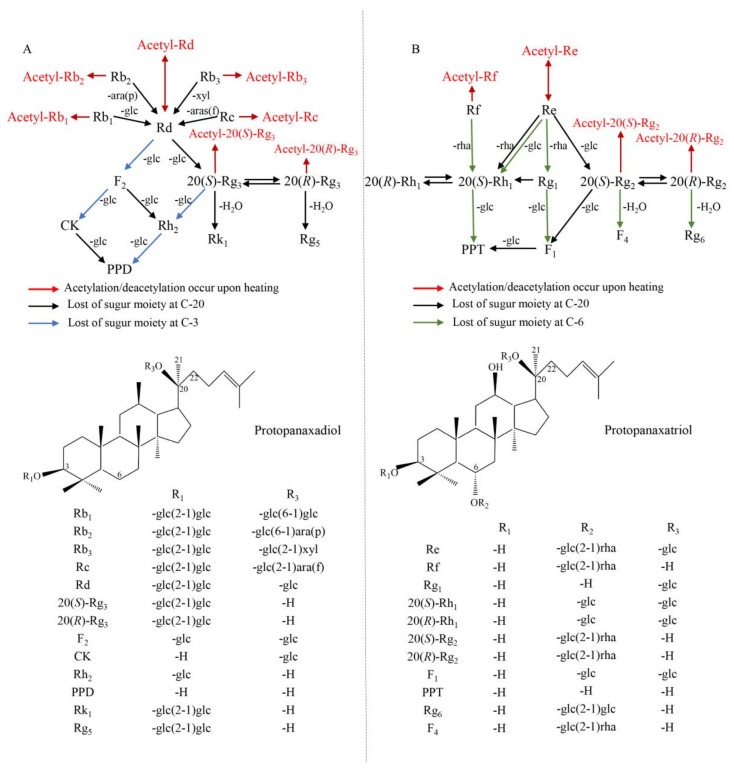
Chemical structures and possible transformation mechanisms of ginsenosides in heated ginseng flowers. (**A**) Protopanaxadiols; (**B**) Protopanaxatriols. Ara(f), α-l-arabinofuranosyl; Ara(p), α-l-arabinopyranosyl; Glc, α-d-glucopyranosyl; Xyl, β-l-xylopyranosyl; Rha, α-l-ahamnopyranosyl. Chemical links between C-20 and C-22 of F_4_ and Rg_5_, and between C-20 and C-21 of Rg_6_ and Rk_1_ are double bonds.

In summary, the 64 ginsenosides identified by the current work have three outcomes. Firstly, 54 ginsenosides, with basic fragment ions at *m*/*z* 459.38 or 475.37, could be grouped into either protopanaxadiol or protopanaxatriol ginsenosides. Except for 20(*R*)-PPT and 20(*S*)-PPT, all the other 62 ginsenosides underwent cleavage of the sugar moiety (or moieties) at C-3 and/or C-20 (protopanaxadiols) and at C-6 and/or C-20 (protopanaxatriols) from their corresponding protopanaxadiol or protopanaxatriol parent structure (for further information, see Figure 4 below). The cleaved sugar moiety (moieties) could be recognized by losing a molecular mass of 162 (-Glc), 146 (-Rha), or 132 (-Ara or -Xyl) Da from the measured value [29]. Secondly, we identified five pairs of enantiomers [20(*R*)/20(*S*)-Rg2, 20(*R*)/20(*S*)-Rh1, 20(*R*)/20(*S*)-Rg3, 20(*R*)/20(*S*)-PPT, and 20(*R*)/20(*S*)-acetyl Rg3] based on the fact that retention time of an individual 20(*S*) ginsenoside is slightly shorter than that of its corresponding 20(*R*) ginsenoside [6,29]. Finally, 23 acetylated ginsenosides [acetyl Re, acetyl Re isomer I, acetyl Re isomer II acetyl Re isomer III, acetyl Re isomer IV, acetyl Re isomer V, acetyl Rg_1_ isomer I, acetyl Rg_1_ II, acetyl Rb_2_, acetyl Rb_2_ isomer, acetyl Rg_3_ isomer, acetyl Rb_3_, acetyl Rd, acetyl Rd isomer I, acetyl Rd isomer II, acetyl Rd isomer III, acetyl-20(*S*)-Rg_3_, acetyl-20(*R*)-Rg_3_, acetyl-Rg_3_ isomer and Rs_1_, Rs_2_, Rs_4_, and Rs_5_] were identified by a loss of 42 Da acetyl groups from their corresponding molecular ions.

### 2.4. Transformation Mechanisms of Ginsenosides in Ginseng Flowers upon Heating

Although several reports have mentioned transformation mechanisms of ginsenosides during the processing of ginseng root, they focused only on changes of ginsenosides after steaming at 100 °C, and none of them dealt with either steamed or baked ginseng flowers as well as transformation of acetylginsenosides. In order to uncover the mechanisms underlying the heat-induced chemical changes in ginsenosides of ginseng flowers, 20 representative ginsenosides were quantified in all the heated ginseng flowers at different temperatures and for various durations as described above.

In principle, the transformation mechanisms among ginsenosides during heating can be extrapolated from their chemical structures, especially changes in number and/or position of their sugar moieties. As shown in Figure 4A, ginsenoside Rd could be formed by hydrolyzing one Glc-, Ara(p)-, Xyl- or Ara(f)-residue attached to C-20 of Rb_1_, Rb_2_, Rb_3_ and Rc, then it further became F_2_ and 20(*S*)-Rg_3_ by losing one Glc-residue attached to its C-3 and C-20, respectively. Further hydrolyzing one more Glc-at C-20 of F_2_ and 20(*S*)-Rg_3_ produced CK or Rh_2_ and Rh_2_ respectively, and both CK and Rh_2_ were finally converted to PPD by losing one Glc-residue. Furthermore, 20(*S*)-Rg_3_ could also be isomerized to 20(*R*)-Rg_3_, and they could be dehydrated at C-20 to yield Rk_1_ and Rg_5_, respectively. Moreover, Rb_1_, Rb_2_, Rb_3_, Rc, 20(*S*)-Rg_3_, 20(*R*)-Rg_3_ and Rd could be acetylated to yield their corresponding acetylginsenosides. In particular, Rd acetylation and deacetylation were reversible.

For the protopanaxatriols shown in Figure 4B, changes of Re to 20(*S*)-Rh_1_, Rg_1_ and 20(*S*)-Rg_2_, Rf and Rg_1_ to 20(*S*)-Rh_1_ (or its 20(*R*)-Rh_1_), Rg_1_ and 20(*S*)-Rg_2_ to F_1_, and finally F_1_ and 20(*S*)-Rh_1_ to 20(*S*)-PPT were occurred via losing one (or two) sugar moiety (moieties) as indicated. Furthermore, 20(*S*)-Rg_2_ and 20(*R*)-Rg_2_ were isomers and became F_4_ and Rg_6_, respectively, by losing one H_2_O. Similar to protopanaxadiols shown Figure 4A, Rf, 20(*S*)-Rg_2_, 20(*R*)-Rg_2_ and Re could be acetylated to yield their corresponding acetylginsenosides, and Re acetylation and deacetylation were also reversible.

To emphasize, it is found for the first time that both the heating methods triggered ginsenoside acetylation and caused the formation of 16 corresponding acetylginsenosides. Based on these findings we generalize the above new transformation mechanism concerning the formation of acetyl-ginsenosides.

As mentioned above, similar results have been partially mentioned in several previous reports. Xu et al. [20] steamed ginseng root at 100 and 120 °C and found that polar ginsenosides were abundant at the lower temperature, and less polar ginsenosides at the higher temperature. Liu et al. [26] compared steamed with non-steamed ginseng roots and found that malonylginsenosides were degraded into their corresponding neutral ginsenosides and that seven polar ginsenosides (Rb_1_, Rb_2_, Rc, Ro, Re, Rg_1_ and Rd) decreased sharply and almost disappeared after steaming for 4 h. Kim et al. [27] reported that three less polar ginsenosides (F_4_, Rg_3_, and Rg_5_) were absent in raw ginseng root, but were detected in ginseng root steamed at 100 °C. All those reports revealed that polar ginsenosides under acidic water solution and high temperature conditions when they were steamed would undergo acid hydrolysis, removing glycosyl(s) to form less polar ginsenosides. However, till now, no one has studied on those conversions of ginsenosides upon baking. Difference between steaming and baking was whether water vapor participated in these reactions or not. Our results showed that ginsenoside transformation also occurred upon baking, although its efficiency was much lower than that observed upon steaming. Therefore, we speculate that water might participate in the transformation via hydration during steaming, and baking treatment could lead transformation of polar to less polar ginsenosides through dehydration. All in all, upon heating, polar ginsenosides could be transformed into less polar ginsenosides. Different from those previous studies, malonyl-ginsenosides were not detected in our study. On the contrary, three acetylginsenosides (acetyl-Re, acetyl-Re isomer V and acetyl-Rd) were identified from UGF, and 20 from heated ginseng flowers in the present work. According to previous reports [26,28], malonyginsenosides could generate malonic acid and acetic acid during steaming at 120 °C for 2 h, and thus could supply an acetate moiety and facilitate acetylation of the glycosyl moiety attached at C-3-OH of seven protopanaxadiols [Rb_1_, Rb_2_, Rb_3_, Rc, Rd and 20(*S*)/20(*R*)-Rg_3_] and at C-6-OH of four protopanaxatriols (Rf, Re, 20(*S*)-Rg_2_ and 20(*R*)-Rg_2_), thus 11 acetylated ginsenosides were identified in the present work, including acetyl-Rb_1_, acetyl-Rb_2_, acetyl-Rb_3_, acetyl-Rc, acetyl-Rd, 20(*S*)-acetyl-Rg_3_, 20(*R*)-acetyl-Rg_3_, acetyl-Rf, acetyl-Re, 20(*S*)-acetyl-Rg_2_, and 20(*R*)-acetyl-Rg_2_.

## 3. Materials and Methods

### 3.1. Chemicals and Plant Materials

HPLC grade acetonitrile was purchased from Fisher Scientific (Pittsburgh, PA, USA). Ultra-pure water was prepared using a Milli-Q50 SP Reagent Water System (Millipore Corporation, Billerica, MA, USA). Other reagents (analytical grade) were purchased from Sinopharm Chemical Reagent Co. Ltd. (Beijing, China). Authentic standard ginsenosides Rh_2_, 20(*S*)-Rg_2_, F_1_, Rd, Rb_1_, Rb_2_, Rf, 20(*S*)-Rg_1_, Rb_3_, Rc, 20(*S*)-Rh_1_, 20(*R*)-Rh_1_, 20(*R*)-Rg_2_, 20(*S*)-Rg_3_, 20(*R*)-Rg_3_, CK, 20(*S*)-PPT and Re were purchased from Lyle Biological (Luoyang, China), and F_4_ and Rg_5_ were bought from Mansite Biological (Chengdu, China). Samples of 3-year-old ginseng flowers were collected on 25 June 2016 and purchased in Huanren County (Benxi, Jilin, China). All samples were morphologically authenticated as the flowers of *P. ginseng* Meyer by Dr. Zhong-hua Liu (Beijing Forest University, Beijing, China). Voucher specimens of *P. ginseng* Meyer were deposited in the Pharmacognosy Laboratory, Shenyang Pharmaceutical University (Shenyang, China). Ginseng flowers were air-dried in the shade, ground and stored at −20 °C for further use.

### 3.2. Crude Extract Preparation

Ground ginseng flower (10.0000 g) in a vessel were baked in an oven at 100, 120, 150 or 180 °C for 0, 1, 2, 4 or 6 h; or were steamed in an autoclave at 100 or 120 °C for 0, 1, 2, 4 or 6 h. After these heating treatments, each of the unheated (0 h) or heated flowers was ultrasonically extracted with 100 mL of 70% aqueous ethanol at 30 °C for 30 min, and the mixture was kept still to obtain the supernatant. The residue was subjected to ultrasonic extraction twice more with the same volume of solvent, and all three supernatants were combined and filtered with No.1 filter paper (Whatman, Maidstone, UK). After filtration, the filtrate was rotary evaporated and dried in a water bath at 40 °C to prepare the crude extract, and the crude extract was stored at −20 °C until use. Supplementary Figure S1 shows a simplified diagram of these experiments.

### 3.3. HPLC Analyses of Ginsenosides

HPLC analyses were performed using a Shimadzu HPLC system (Shimadzu, Kyoto, Japan) equipped with two LC-10AT VP pumps, a SPDM20A ultraviolet detector, and a SIL-20AC TH autosampler controlled by an analytical software package (LC Solution-Release 1.23SP1). A reversed phase column (Diamonsil C_18_ 5 μm 250 × 4.6 mm i.d., Dikma, Beijing, China) was used for separation, and the column temperature was set at 25 °C. The solvent system consisted of water (A) and acetonitrile (B) under the following gradient program: 0 min, 21% B; 0–14 min, 21% B; 14–24 min, 30% B; 24–55 min, 32% B; 55–75 min, 33% B; 75–100 min, 35% B; 100–120 min, 37% B; 120–130 min, 60% B; 130–140 min, 70% B; 140–150 min, 80% B. The flow rate was set at 0.8 mL/min with an injection volume of 10 µL. Detection wavelength was set at 203 nm to monitor more ginsenosides simultaneously.

To determine the ginsenosides in unheated, baked and steamed ginseng flowers, a stock solution of the 20 mixed authentic standards was prepared, and injected with six volumes (2, 4, 6, 10, 16, 20 μL) for their linearity assessments. Linearities were established within the range of 0.88–10.8 μg and good linearity (*R*^2^ > 0.999) and high precision, stability and repeatability (with all their relative standard deviations being less than 5%) was obtained for each of the 20 ginsenosides. The standard curves and regression equations of the 20 authentic standard ginsenosides are shown in Supplementary Figure S2.

### 3.4. UPLC-QTOF-MS/MS Analyses of Ginsenosides

The UPLC-QTOF-MS/MS system comprised an Acquity Ultra-Performance Liquid Chromatography (UPLC) system and a Xevo G2-XS type QTOF-MS mass spectrometer (Waters, Milford, MA, USA). Each of the crude extracts prepared above was dissolved at concentration of 1 mg/mL in chromatographic pure methanol, and the dissolved solution was filtered through a 0.2 μm membrane filter prior to the analysis with the UPLC, in which the reversed phase column was also used, and the column temperature was also set at 25 °C. Mass spectra were recorded within the range of *m*/*z* 100–1500 in both positive and negative ionization modes under the following conditions: nitrogen drying gas flow, 10.0 L/min; nebulizer pressure, 45 psi; gas drying temperature, 370 °C; capillary and fragment or voltage, 2.500 kV; and MS/MS collision energies, 20 V.

### 3.5. Statistical Analysis

Statistical significance (*t*-test: two-sample equal variance, using two-tailed distribution) was determined by using the Microsoft Excel statistical software (Microsoft Office Excel 2016, Microsoft Corp., Redmond, WA, USA). *p* < 0.05 was set to be significant. Correlations were analyzed using SPSS for Windows (Version 17.0, SPSS Inc., Chicago, IL, USA) and calculated using the correlation coefficient statistical option in the Pearson test. All data were presented as mean ± S.D. of three parallel measurements.

## Figures and Tables

**Table 1:** Identification of ginsenosides by UPLC-Q-TOF/MS in ginseng flowers before and after baking and steaming flowers.

No.	Rt (min)	Compound Name	Error (ppm)	Molecular Formula	Measured Value (*m*/*z*)	MS/MS Fragments *m*/*z*	Sample Items
**1**	20.96	Rg_1_	0.8	C_42_H_72_O_14_	799.48	637.43[M-(Glu-H_2_O)-H]^−^/475.38[M-2(Glu-H_2_O)-H]^−^	All
**2**	21.19	Re	1.1	C_48_H_82_O_18_	945.57	783.49[M-(Glu-H_2_O)-H]^−^/637.43[M-(Glu-H_2_O)-(Rha-H_2_O)-H]^−^/475.38[M-2(Glu-H_2_O)-(Rha-H_2_O)-H]^−^	All
3	22.98	G-Ia	−1.9	C_42_H_72_O_14_	799.49	637.43[M-(Glu-H_2_O)-H]^−^/475.32[M-2(Glu-H_2_O)-H]^−^	S1206
4	24.16	Re isomer	−0.6	C_48_H_82_O_18_	945.55	799.49[M-(Rha-H_2_O)-H]^−^/637.43[M-(Glu-H_2_O)-(Rha-H_2_O)-H]^−^/475.33[M-2(Glu-H_2_O)-(Rha-H_2_O)-H]^−^	S1206
**5**	26.69	Floral G-P	−2.4	C_53_H_90_O_23_	1093.58	961.53[M-(Ara-H_2_O)-H]^−^/799.48[M-(Ara-H_2_O)-(Glu-H_2_O)-H]^−^/637.43[M-(Ara-H_2_O)-2(Glu-H_2_O)-H]^−^/ 475.37[M-(Ara-H_2_O)-3(Glu-H_2_O)-H]^−^	UPG, B1206, B1506
6	27.80	Acetyl-Re isomer I	0.4	C_50_H_84_O_19_	987.60	945.54[M-Ac-H]^−^/799.48[M-Ac-(Rha-H_2_O)-H]^−^/637.43[M-Ac-(Glu-H_2_O)-(Rha-H_2_O)-H]^−^/ 475.38[M-Ac-2(Glu-H_2_O)-(Rha-H_2_O)-H]^−^	B1506
**7**	28.52	Floral G-P isomer	−1.3	C_53_H_90_O_23_	1093.58	961.54[M-(Ara-H_2_O)-H]^−^/799.48[M-(Ara-H_2_O)-(Glu-H_2_O)-H]^−^/637.41[M-(Ara-H_2_O)-2(Glu-H_2_O)-H]^-^	UPG, S1204
8	28.72	Acetyl-Re isomer II	−3.2	C_50_H_84_O_19_	987.56	945.54[M-Ac-H]^−^/799.48[M-Ac-(Ara-H_2_O)-(Glu-H_2_O)-H]^−^/637.43[M-Ac-(Glu-H_2_O)-(Rha-H_2_O)-H]^−^/ 475.30[M-Ac-(Glu-H_2_O)-(Rha-H_2_O)-H]^−^	B1206, B1506
9	28.89	Acetyl-Re isomer III	−1.4	C_50_H_84_O_19_	987.56	945.54[M-Ac-H]^−^/799.48[M-(Ara-H_2_O)-(Glu-H_2_O)-H]^−^/637.43[M-Ac-(Glu-H_2_O)-(Rha-H_2_O)-H]^−^/ 475.38[M-Ac-(Glu-H_2_O)-(Rha-H_2_O)-H]^−^	B1506
10	29.73	Acetyl- Rg_1_I/isomer	−1.5	C_44_H_74_O_15_	841.56	799.48[M-Ac-H]^−^/637.43[M-Ac-(Glu-H_2_O)-H]^−^/475.37[M-Ac-(Glu-H_2_O)-(Rha-H_2_O)-H]^−^	B1506
11	30.57	Acetyl-Re isomer IV	−0.7	C_50_H_84_O_19_	987.58	945.54[M-Ac-H]^−^/799.48[M-(Ara-H_2_O)-(Glu-H_2_O)-H]^−^/637.43[M-Ac-(Glu-H_2_O)-(Rha-H_2_O)-H]^−^/ 475.38[M-Ac-(Glu-H_2_O)-(Rha-H_2_O)-H]^−^	B1506
**12**	31.55	G-Re_1_/_2_/_3_/NG-N isomer	0.9	C_48_H_82_O_19_	961.56	799.49[M-(Glu-H_2_O)-H]^−^/637.43[M-2(Glu-H_2_O)-H]^−^/475.38[M-3(Glu-H_2_O)-H]^−^	All
**13**	32.89	Acetyl-Re	0.2	C_50_H_84_O_19_	987.55	945.54[M-Ac-H]^−^/927.5[M-Ac-OH]^−^/799.48[M-(Ara-H_2_O)-(Glu-H_2_O)-H]^−^/637.43[M-(Ara-H_2_O)-2(Glu-H_2_O)-H]^−^	All
14	33.18	Acetyl-Rg_1_ II	1	C_44_H_74_O_15_	841.56	799.48[M-Ac-H]^−^/637.43[M-Ac-(Glu-H_2_O)-H]^−^/475.37[M-Ac-(Ara-H_2_O)-(Glu-H_2_O)-H]^−^	B1506
**15**	36.52	Rf	−0.1	C_42_H_72_O_14_	799.49	637.43[M-(Glu-H_2_O)-H]^−^/475.38[M-2(Glu-H_2_O)-H]^−^	All
**16**	39.86	NG-R_2_	−1.2	C_41_H_70_O_13_	769.48	637.43[M-(Ara-H_2_O)-H]^−^/475.38[M-(Ara-H_2_O)-(Glu-H_2_O)-H]^−^	All
**17**	41.00	F_5_/F_3_	−1.2	C_41_H_70_O_13_	769.47	637.43[M-(Ara-H_2_O)-H]^−^/475.38[M-(Ara-H_2_O)-(Glu-H_2_O)-H]^−^	All
**18**	42.90	Rb_1_	−0.3	C_54_H_92_O_23_	1107.62	945.54[M-(Glu-H_2_O)-H]^−^/783.48[M-2(Glu-H_2_O)-H]^−^/621.43[M-3(Glu-H_2_O)-H]^−^/459.38[M-4(Glu-H_2_O)-H]^−^	All
19	42.89	F_5_/F_3_	0.4	C_41_H_70_O_13_	769.48	637.42[M-(Rha-H_2_O)-H]^−^/475.37[M-(Rha-H_2_O)-(Glu-H_2_O)-H]^−^	S1204, B1206, B1506
**20**	43.23	20(*S*)-Rg_2_	0.8	C_42_H_71_O_13_	783.49	637.43[M-(Rha-H_2_O)-H]^−^/475.38[M-(Rha-H_2_O)-(Glu-H_2_O)-H]^−^	All
21	44.57	20(*S*)-Rh_1_	−0.6	C_36_H_62_O_9_	637.43	475.38[M-(Glu-H_2_O)-H]^−^	S1204, S1206, B1206
22	45.37	20(*R*)-Rg_2_	0.8	C_42_H_71_O_13_	783.49	637.43[M-(Rha-H_2_O)-H]^−^/475.38[M-(Rha-H_2_O)-(Glu-H_2_O)-H]^−^	S1204, S1206
**23**	47.01	Rc	−0.8	C_53_H_90_O_22_	1077.60	945.54[M-(Ara-H_2_O)-H]^−^/783.49[M-(Ara-H_2_O)-(Glu-H_2_O)-H]^−^/621.44[M-(Ara-H_2_O)-2(Glu-H_2_O)-H]^−^/ 459.38[M-(Ara-H_2_O)-3(Glu-H_2_O)-H]^−^	All
24	47.29	20(*R*)-Rh_1_	2.2	C_36_H_62_O_9_	637.44	475.3811[M-(Glu-H_2_O)-H]^−^	S1204, S1206
25	49.47	PQ-R_1_	−1.5	C_56_H_94_O_24_	1149.76	1107.59[M-Ac-H]^−^/945.54[M-Ac-(Glu-H_2_O)-H]^−^/783.49[M-Ac-2(Glu-H_2_O)-H]^−^/ 621.43[M-Ac-3(Glu-H_2_O)-H]^−^/459.38[M-Ac-4(Glu-H_2_O)-H]^−^	B1506
**26**	52.17	Rb_2_	−0.7	C_53_H_90_O_22_	1077.60	945.54[M-(Ara(p)-H_2_O)-H]^−^/783.49[M-(Ara(p)-H_2_O)-(Glu-H_2_O)-H]^−^/ 621.44[M-(Ara(p)-H_2_O)-2(Glu-H_2_O)-H]^−^/459.38[M-(Ara(p)-H_2_O)-3(Glu-H_2_O)-H]^−^	All
**27**	54.08	Rb_3_	−1.5	C_53_H_90_O_22_	1077.59	945.54[M-(Xyl-H_2_O)-H]^−^/783.49[M-(Xyl(p)-H_2_O)-(Glu-H_2_O)-H]^−^/ 621.44[M-(Xyl(p)-H_2_O)-2(Glu-H_2_O)-H]^−^/459.37[M-(Xyl(p)-H_2_O)-3(Glu-H_2_O)-H]^−^	All
**28**	56.46	F_1_	1.6	C_36_H_62_O_9_	637.44	475.37[M-(Glu-H_2_O)-H]^−^	All
29	57.91	Acetyl-Re isomer V	−1.4	C_51_H_84_O_21_	987.56	945.54[M-Ac-H]^−^/783.48[M-Ac-(Glu-H_2_O)-H]^−^/637.43[M-(Glu-H_2_O)-(Rha-H_2_O)-H]^−^/ 475.38[M-2(Glu-H_2_O)-(Rha-H_2_O)-H]^−^	UPG
30	59.09	PQ-R_1_ isomer	−0.3	C_56_H_94_O_24_	1149.81	1107.59[M-Ac-H]^−^/945.54[M-Ac-(Glu-H_2_O)-H]^−^/783.49[M-Ac-2(Glu-H_2_O)-H]^−^/ 621.43[M-Ac-3(Glu-H_2_O)-H]^−^/475.3767[M-Ac-(Rha-H_2_O)-3(Glu-H_2_O)-H]^−^	S1204, S1206, B1206, B1506
31	61.46	Acetyl-Rb_2_ isomer	−0.1	C_55_H_92_O_23_	1119.71	1077.58[M-Ac-H]^−^/945.54[M-Ac-(Ara(p)-H_2_O)-H]^−^/783.48[M-Ac-(Ara(p)-H_2_O)-(Glu-H_2_O)-H]^−^/ 621.43[M-Ac-(Ara(p)-H_2_O)-2(Glu-H_2_O)-H]^−^/459.38[M-Ac-(Ara(p)-H_2_O)-3(Glu-H_2_O)-H]^−^	B1506
**32**	64.88	Rd	−0.3	C_48_H_82_O_18_	945.56	783.49[M-(Glu-H_2_O)-H]^−^/621.44/459.38[M-2(Glu-H_2_O)-H]^−^	All
33	65.51	Rs_1_/Rs_2_	−2.8	C_55_H_92_O_23_	1119.75	1077.5[M-Ac-H]^−^/945.53[M-Ac-(Ara(p)-H_2_O)-H]^−^/783.48[M-Ac-(Ara(p)-H_2_O)-(Glu-H_2_O)-H]^−^/ 621.43[M-Ac-(Ara(p)-H_2_O)-2(Glu-H_2_O)-H]^−^/459.38[M-Ac-(Ara(p)-H_2_O)-3(Glu-H_2_O)-H]^−^	B1206, B1506
34	70.40	Acetyl-Rg_3_ isomer	0.1	C_44_H_74_O_14_	825.51	783.49[M-Ac-H]^−^/637.43[M-Ac-(Rha-H_2_O)-H]^−^/475.37[M-Ac-(Rha-H_2_O)-(Glu-H_2_O)-H]^−^	B1506
35	73.77	Rs_1_/Rs_2_	−0.8	C_55_H_92_O_23_	1119.78	1077.58[M-Ac-H]^−^/945.54[M-Ac-(Ara(p)-H_2_O)-H]^−^/783.49[M-Ac-(Ara(p)-(Glu-H_2_O)-H_2_O)-H]^−^/ 621.43[M-Ac-(Ara(p)-2(Glu-H_2_O)-H_2_O)-H]^−^/459.37[M-Ac-(Ara(p)-3(Glu-H_2_O)-H_2_O)-H]^−^	S1204, S1206, B1206, B1506
36	73.96	PQ-R_1_ isomer	−1.5	C_56_H_94_O_24_	1149.78	1107.59[M-Ac-H]^−^/1089.58[M-Ac-OH]^−^/945.53[M-Ac-(Glu-H_2_O)-H]^−^/783.49[M-Ac-2(Glu-H_2_O)-H]^−^/ 621.43[M-Ac-3(Glu-H_2_O)-H]^−^/459.38[M-Ac-4(Glu-H_2_O)-H]^−^	B1206
**37**	82.72	Vinaginsenoside R_16_	−2.5	C_47_H_80_O_17_	915.53	753.48[M-(Glu-H_2_O)-H]^−^/621.44[M-(Ara(p)-H_2_O)-(Glu-H_2_O)-H]^−^/ 475.37[M-(Ara(p)-H_2_O)-(Glu-H_2_O)-(Rha-H_2_O)-H]^−^	UPG, S1204
38	75.82	Acetyl-Rb_2_	−2	C_55_H_92_O_23_	1119.77	1077.58[M-Ac-H]^−^/945.54[M-Ac-(Ara(p)-H_2_O)-H]^−^/783.48[M-Ac-(Ara(p)-H_2_O)-(Glu-H_2_O)-H]^−^/ 621.43[M-Ac-(Ara(p)-H_2_O)-2(Glu-H_2_O)-H]^−^/459.38[M-Ac-(Ara(p)-H_2_O)-3(Glu-H_2_O)-H]^−^	B1206, B1506
39	78.02	Acetyl-Rd isomer I	−1.1	C_50_H_84_O_19_	987.67	945.54[M-Ac-H]^−^/783.48[M-Ac-(Glu-H_2_O)-H]^−^/621.43[M-Ac-2(Glu-H_2_O)-H]^−^/459.38[M-Ac-3(Glu-H_2_O)-H]^−^	B1206, B1506
40	79.54	Acetyl-Rb_3_	−1.5	C_55_H_92_O_23_	1119.76	1077.58[M-Ac-H]^−^/1059.57[M-Ac-OH]^−^/945.54[M-Ac-(Ara(p)-H_2_O)-H]^−^/ 783.49[M-Ac-(Ara(p)-H_2_O)-2(Glu-H_2_O)-H]^−^/621.43[M-Ac-(Ara(p)-H_2_O)-3(Glu-H_2_O)-H]^−^/ 459.38[M-Ac-(Ara(p)-H_2_O)-4(Glu-H_2_O)-H]^−^	B1206, B1506
41	81.22	Acetyl-Rd isomer II	−3.9	C_50_H_84_O_19_	987.50	945.54[M-Ac-H]^−^/799.48[M-Ac-(Rha(p)-H_2_O)-H]^−^/621.43[M-Ac-(Rha(p)-H_2_O)-(Glu-H_2_O)-OH]^−^/ 459.38[M-Ac-(Rha(p)-H_2_O)-2(Glu-H_2_O)-OH]^−^	B1206, B1506
**42**	92.13	Acetyl-Rd	1.4	C_50_H_84_O_19_	987.67	945.54[M-Ac-H]^−^/927.52[M-Ac-OH]^−^/783.49[M-Ac-(Glu-H_2_O)-H]^−^/621.44[M-Ac-2(Glu-H_2_O)-H]^−^/ 459.37[M-Ac-3(Glu-H_2_O)-H]^−^	All
**43**	95.90	NG-Fe	−0.4	C_47_H_80_O_17_	915.53	783.43[M-(Ara-H_2_O)-H]^−^/621.43[M-(Ara-H_2_O)-(Glu-H_2_O)-H]^−^/459.37[M-(Ara-H_2_O)-2(Glu-H_2_O)-H]^−^	UPG, S1204, S1206
44	98.60	Acetyl-Rd isomer III	−1.1	C_50_H_84_O_19_	987.71	945.54[M-Ac-H]^−^/783.43[M-Ac-(Glu-H_2_O)-H]^−^/621.43[M-Ac-2(Glu-H_2_O)-H]^−^/459.38[M-Ac-3(Glu-H_2_O)-H]^−^	B1206, B1506
45	100.46	Rg_6_ isomer	0	C_42_H_69_O_12_	765.48	619.42[M-(Rha-H_2_O)-H]^−^/457.36[M-(Rha-H_2_O)-(Glu-H_2_O)-H]^−^	B1506
46	101.46	Rg_6_	−0.8	C_42_H_69_O_12_	765.48	619.42[M-(Rha-H_2_O)-H]^−^/457.37[M-(Rha-H_2_O)-(Glu-H_2_O)-H]^−^	S1204, S1206, B1506
47	104.98	Pseudo-G-RC_1_	−1	C_50_H_84_O_19_	987.43	945.54[M-Ac-H]^−^/783.48[M-Ac-(Glu-H_2_O)-H]^−^/621.43[M-Ac-2(Glu-H_2_O)-H]^−^/459.38[M-Ac-3(Glu-H_2_O)-H]^−^	S1204, S1206, B1206, B1506
48	109.06	F_4_	−1.2	C_42_H_69_O_12_	765.48	619.41[M-(Rha-H_2_O)-H]^−^/457.37[M-(Rha-H_2_O)-(Glu-H_2_O)-H]^−^	S1204, S1206, B1206, B1506
49	109.23	Rk_3_	1.1	C_36_H_59_O_8_	619.42	457.36[M-(Glu-H_2_O)-H]^−^	S1206
50	114.46	Rg_3_ isomer	1.3	C_42_H_72_O_13_	783.69	621.44[M-(Glu-H_2_O)-H]^−^/459.38[M-2(Glu-H_2_O)-H]^−^	S1204
51	116.00	G-La	0.3	C_42_H_69_O_13_	781.47	619.42[M-(Glu-H_2_O)-H]^−^/455.34[M-2(Glu-H_2_O)-H]^−^	S1206
52	118.43	20(*S*)-Rg_3_	0.1	C_42_H_72_O_13_	783.49	621.44[M-(Glu-H_2_O)-H]^−^/459.38[M-2(Glu-H_2_O)-H]^−^	S1204, S1206, B1206, B1506
53	119.01	20(*R*)-Rg_3_	0.1	C_42_H_72_O_13_	783.49	621.44[M-(Glu-H_2_O)-H]^−^/459.38[M-2(Glu-H_2_O)-H]^−^	S1204, S1206, B1206
54	121.37	20(*S*)-PPT	0.6	C_30_H_52_O_4_	475.38	475.38	S1204, S1206
55	121.94	20(*R*)-PPT	−1.9	C_30_H_52_O_4_	475.37	475.37	S1204, S1206
56	122.26	Acetyl-20(*S*)-Rg_3_	−0.1	C_44_H_74_O_14_	825.50	783.49[M-Ac-H]^−^/765.48[M-Ac-OH]^−^/621.44[M-Ac-(Glu-H_2_O)-H]^−^/459.38[M-Ac-2(Glu-H_2_O)-H]^−^	S1204, S1206, B1506
57	122.75	Acetyl-20(*R*)-Rg_3_	−0.4	C_44_H_74_O_14_	825.50	783.49[M-Ac-H]^−^/765.48[M-Ac-OH]^−^/621.44[M-Ac-(Glu-H_2_O)-H]^−^/459.38[M-Ac-2(Glu-H_2_O)-H]^−^	S1204, S1206, B1506
58	123.72	Acetyl-Rg_3_ isomer	0.2	C_44_H_74_O_14_	825.50	783.49[M-Ac-H]^−^/459.36[M-Ac-2(Glu-H_2_O)-H]^−^	S1204, S1206
59	124.73	Rk_1_	0.5	C_42_H_69_O_12_	765.49	603.44[M-(Glu-H_2_O)-H]^−^	S1204, S1206, B1206, B1506
60	125.41	Rg_5_	0.5	C_42_H_69_O_12_	765.49	603.43[M-(Glu-H_2_O)-H]^-^	S1204, S1206, B1506
61	126.59	CK	0.1	C_36_H_62_O_8_	621.44	459.38[M-(Glu-H_2_O)-H]^−^	S1204, S1206, B1506
62	127.99	Rh_2_	2.4	C_36_H_62_O_8_	621.44	459.38[M-(Glu-H_2_O)-H]^−^	S1204, S1206, B1506
63	130.30	Rs_5_	−0.2	C_44_H_71_O_13_	807.50	765.48[M-Ac-H]^−^/603.43[M-Ac-(Glu-H_2_O)-H]^−^	S1204, S1206, B1506
64	131.31	Rs_4_	−0.4	C_44_H_71_O_13_	807.49	765.48[M-Ac-H]^−^/603.43[M-Ac-(Glu-H_2_O)-H]^−^	S1204, S1206, B1506

Data colored in black represent ginsenosides originally existed in UGF, and those in blue represent ginsenosides newly generated in heat-processed ginseng flowers. Numbers in the most left column colored in yellow represent ginsenosides newly generated in S1204 and/or S1206; those in red, ginsenosides newly generated in B1206 and/or B1506; those in brown, ginsenosides newly generated in S1204, S1206, B1206, and B1506; and those in green, ginsenosides existed in UGF but were not detected after heating. UGF, unprocessed ginseng flower; S1204 and S1206, steaming ginseng flowers at 120 °C for 4 and 6 h, respectively; B1206 and B1506, baking ginseng flowers for 6 h at 120 and 150 °C, respectively.

## Supplementary Materials

The following are available online, Figure S1: A simplified protocol for baking and steaming Air-dried ginseng flowers, Figure S2: UPLC chromatograms at 203 nm of the 20 authentic ginsenosides and extracts of unheated ginseng flowers (UGF) and several representative heated ginseng flowers. Shown in sequence from top to bottom are chromatograms of the 20 authentic ginsenosides (A) and extracts of UGF (B), B1206 (C), B1506 (D), S1204 (E), and S1206 (F). Ginsenosides marked are: (1) Rg_1_, (2) Re, (3) Rf, (4) Rb_1_, (5) 20(*S*)-Rg_2_, (6) 20(*S*)-Rh_1_, (7) 20(*R*)-Rg_2_, (8) Rc, (9) 20(*R*)-Rh_1_, (10) Rb_2_, (11) Rb_3_, (12) F_1_, (13) Rd, (14) F_4_, (15) 20(*S*)-Rg_3_, (16) 20(*R*)-Rg_3_, and (17) 20(*S*)-PPT, (18) Rg_5_, (19) CK, and (20) Rh_2_.

## References

[1] Ellis J.M., Reddy P. (2003). The effects of *Panax ginseng* on quality of life. J. Clin. Pharm. Ther..

[2] In G., Ahn N.G., Bae B.S., Lee M.W., Park H.W., Jang K.H., Cho B.G., Han C.K., Park C.K., Kwak Y.S. (2017). In Situ analysis of chemical components induced by steaming between fresh ginseng, steamed ginseng, and red ginseng. J. Ginseng Res..

[3] Xie Y.Y., Luo D., Cheng Y.J., Ma J.F., Wang Y.M., Liang Q.L., Luo G.A. (2012). Steaming-induced chemical transformations and holistic quality assessment of red ginseng derived from *Panax ginseng* by means of HPLC-ESI-MS/MS(n)-based multicomponent quantification fingerprint. J. Agric. Food Chem..

[4] Christensen L.P. (2009). Chapter 1 Ginsenosides. Adv. Food Nutr. Res..

[5] Qi L.W., Wang C.Z., Yuan C.S. (2011). Isolation and analysis of ginseng: Advances and challenges. ChemInform.

[6] Yang H., Lee D.Y., Kang K.B., Kim J.Y., Kim S.O., Yoo Y.H., Sung S.H. (2015). Identification of ginsenoside markers from dry purified extract of *Panax ginseng* by a dereplication approach and UPLC-QTOF/MS analysis. J. Pharm. Biomed. Anal..

[7] Xu T., Jin Z., Yuan Y., Wei H., Xu X., He S., Chen S., Hou W., Guo Q., Hua B. (2016). Ginsenoside Rg3 serves as an adjuvant chemotherapeutic agent and VEGF inhibitor in the treatment of non-small cell lung cancer: A meta-analysis and systematic review. Evid.-Based Complement. Altern. Med..

[8] Yang X.D., Yang Y.Y., Ouyang D.S., Yang G.P. (2015). A review of biotransformation and pharmacology of ginsenoside compound K. Fitoterapia.

[9] Zhang H.M., Li S.L., Zhang H., Wang Y., Zhao Z.L., Chen S.L., Xu H.X. (2012). Holistic quality evaluation of commercial white and red ginseng using a UPLC-QTOF-MS/MS-based metabolomics approach. J. Pharm. Biomed. Anal..

[10] Wang C.Z., Aung H.H., Ni M., Wu J.A., Tong R.B., Wicks S., He T.C., Yuan C.S. (2007). Red American ginseng: Ginsenoside constituents and antiproliferative activities of heat-processed *Panax quinquefolius* roots. Planta Med..

[11] Yun T.K., Lee Y.S., Lee Y.H. (2001). Anticarcinogenic effect of *Panax ginseng C.A.* Meyer and identification of active compounds. J. Korean Med. Sci..

[12] Hong C.E., Lyu S.Y. (2011). Anti-inflammatory and anti-oxidative effects of Korean red ginseng extract in human keratinocytes. Immune Netw..

[13] Wu W., Lu Z., Teng Y., Guo Y., Liu S. (2016). Structural characterization of ginsenosides from flower buds of *Panax ginseng* by RRLC-Q-TOF MS. J. Chromatogr. Sci..

[14] Nguyen H.T., Song G.Y., Kim J.A., Hyun J.H., Kang H.K., Kim Y.H. (2010). Dammarane-type saponins from the flower buds of Panax ginseng and their effects on human leukemia cells. Bioorg. Med. Chem. Lett..

[15] Li S.S., Li K.K., Xu F., Tao L., Yang L., Chen S.X. (2017). A strategy for simultaneous isolation of less polar ginsenosides, including a pair of new 20-methoxyl isomers, from flower buds of *Panax ginseng*. Molecules.

[16] Yoshikawa M., Sugimoto S., Nakamura S. (2007). Medicinal flowers. XI. Structures of new dammarane-type triterpene diglycosides with hydroperoxide group from flower buds of *Panax ginseng*. Chem. Pharm. Bull..

[17] Ko S.K., Cho O.S., Bae H.M., Im B.O., Lee O.H., Lee B.Y. (2011). Quantitative analysis of ginsenosides composition in flower buds of various ginseng plants. J. Korean Soc. Appl. Biol..

[18] Cho K., Song S.B., Tung N.H., Kim K.E., Kim Y.H. (2014). Inhibition of TNF-α-Mediated NF-ĸB transcriptional activity by dammarane-type ginsenosides from steamed flower buds of *Panax ginseng* in HepG2 and SK-Hep1 cells. Biomol. Ther..

[19] Tung N.H., Quang T.H., Son J.H., Koo J.E., Hong H.J., Koh Y.S., Song G.Y., Kim Y.H. (2011). Inhibitory effect of ginsenosides from steamed ginseng-leaves and flowers on the LPS-stimulated IL-12 production in bone marrow-derived dendritic cells. Food Sci. Biotechnol..

[20] Xu X.F., Gao Y., Xu S.Y., Liu H., Xue X., Zhang Y., Zhang H., Liu M.N., Xiong H., Lin R.C. (2017). Remarkable impact of steam temperature on ginsenosides transformation from fresh ginseng to red ginseng. J. Ginseng Res..

[21] Zhou Q.L., Zhu D.N., Yang Y.F., Xu W., Yang X.W. (2017). Simultaneous quantification of twenty-one ginsenosides and their three aglycones in rat plasma by a developed UFLC-MS/MS assay: Application to a pharmacokinetic study of red ginseng. J. Pharm. Biomed. Anal..

[22] Wang H.P., Zhang Y.B., Yang X.W., Zhao D.Q., Wang Y.P. (2016). Rapid characterization of ginsenosides in the roots and rhizomes of *Panax ginseng* by UPLC-DAD-QTOF-MS/MS and simultaneous determination of 19 ginsenosides by HPLC-ESI-MS. J. Ginseng Res..

[23] Du Q.Q., Liu S.Y., Xu R.F., Li M., Song F.R., Liu Z.Q. (2012). Studies on structures and activities of initial maillard reaction products by electrospray ionisation mass spectrometry combined with liquid chromatography in processing of red ginseng. Food Chem..

[24] Li Y., Liu X., Meng L., Wang Y. (2018). Qualitative and quantitative analysis of furosine in fresh and processed ginsengs. J. Ginseng Res..

[25] Qiu S., Yang W.Z., Yao C.L. (2017). Malonyl ginsenosides with potential antidiabetic activities from the flower buds of Panax ginseng. J. Nat. Prod..

[26] Liu Z., Xia J., Wang C.Z., Zhang J.Q., Ruan C.C., Sun G.Z., Yuan C.S. (2016). Remarkable impact of acidic ginsenosides and organic acids on ginsenoside transformation from fresh ginseng to red ginseng. J. Agric. Food. Chem..

[27] Kim W.Y., Kim J.M., Han S.B., Lee S.K., Kim N.D., Park M.K. (2000). Steaming of ginseng at high temperature enhances biological activity. J. Nat. Prod..

[28] Xiao S.Y., Luo G.A. (2005). Chemical reactions of ginsenosides in red ginseng processing by HPLC/MS/MS. Chin. Tradit. Herbal Drugs.

[29] Qi L.W., Wang H.Y., Zhang H., Wang C.Z., Li P., Yuan C.S. (2012). Diagnostic ion filtering to characterize ginseng saponins by rapid liquid chromatography with time-of-flight mass spectrometry. J. Chromatogr. A.

