# Supplementary figures and images for: Intraconversion of Polar Ginsenosides, Their Transformation into Less-Polar Ginsenosides, and Ginsenoside Acetylation in Ginseng Flowers upon Baking and Steaming
Source: Molecules. 2018 Mar 26;23(4):759. doi: 10.3390/molecules23040759 (PMC6017459; doi:10.3390/molecules23040759)

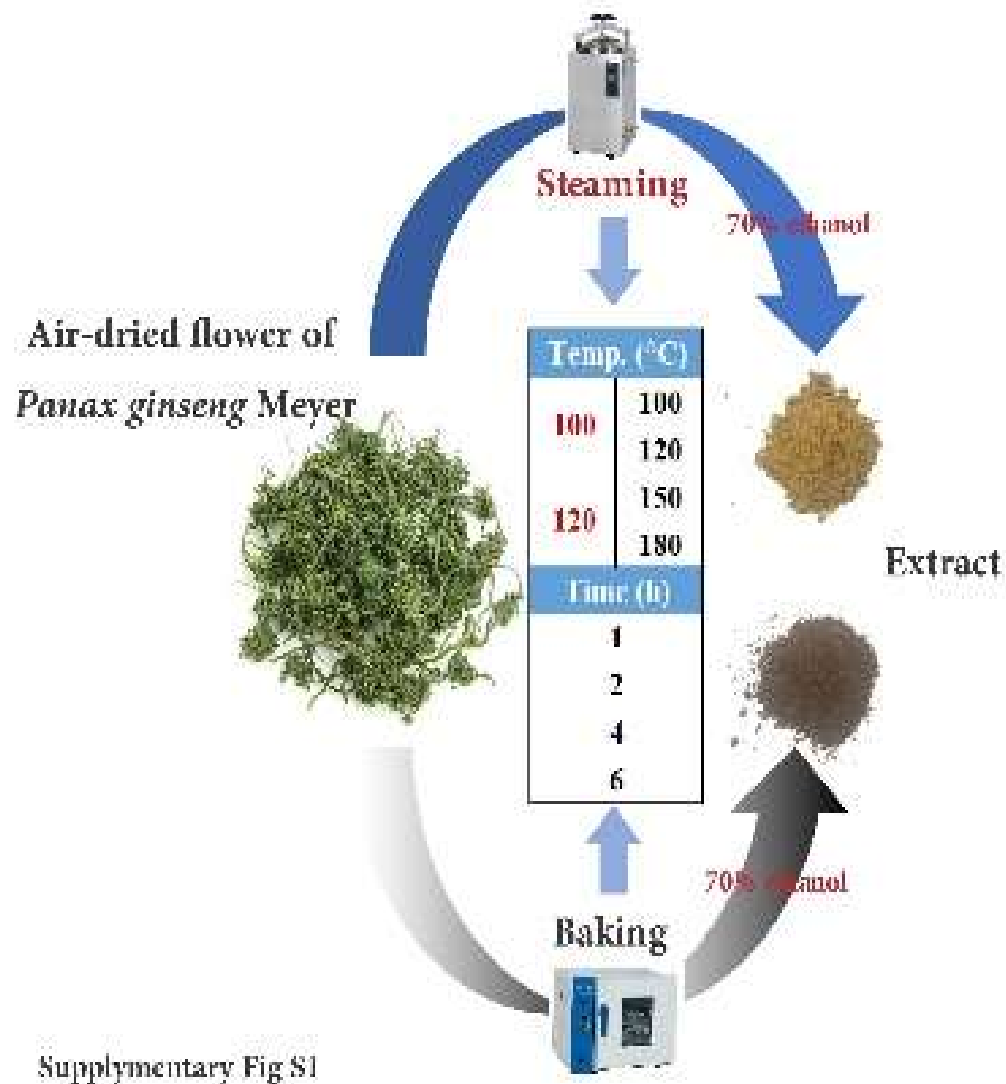

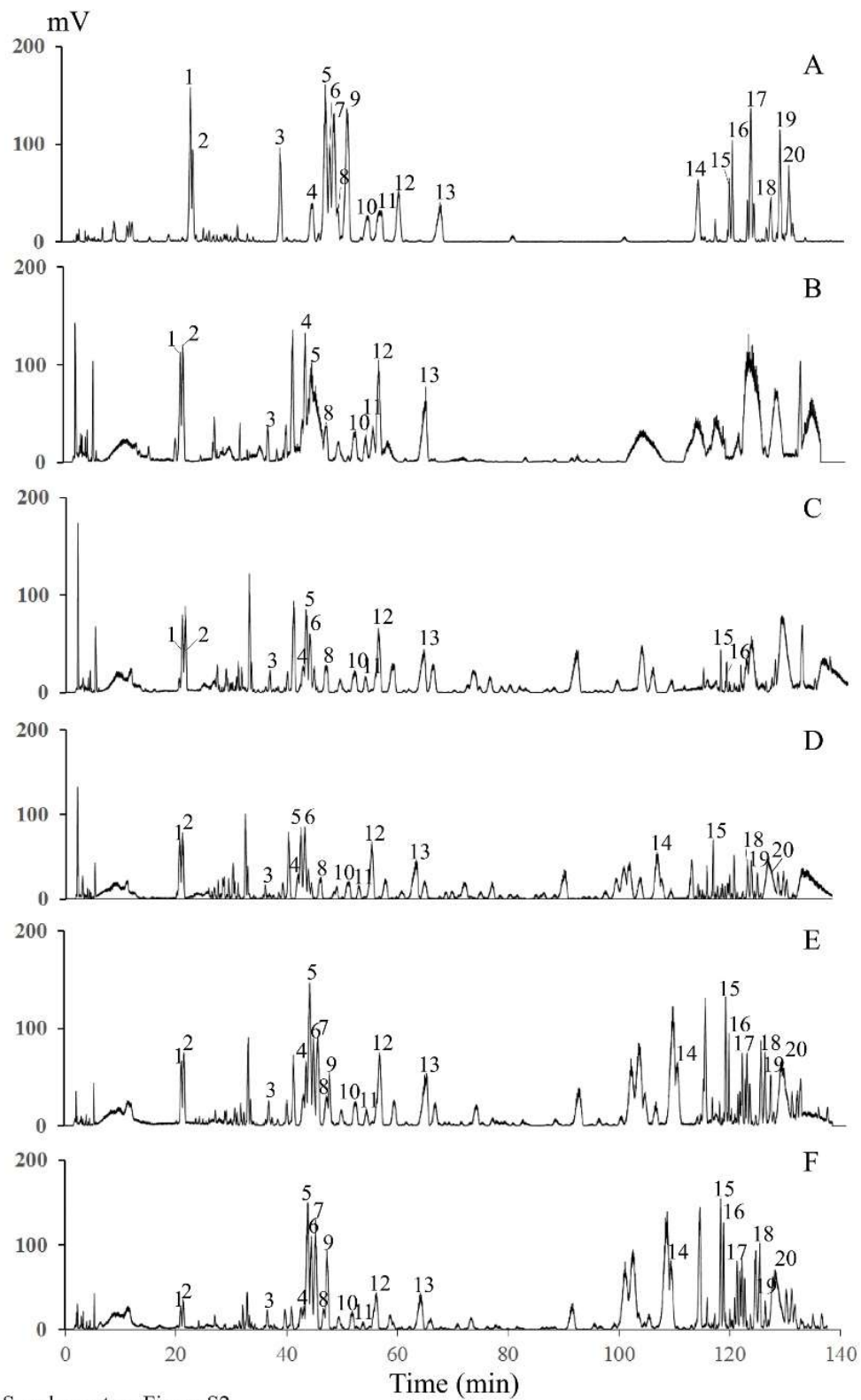

Supplementary Figure S2

Supplement: Supplementary file 1 [file molecules-23-00759-s001.pdf]
